# The genome sequence of the wood white butterfly, *Leptidea sinapis* (Linnaeus, 1758)

**DOI:** 10.12688/wellcomeopenres.18118.1

**Published:** 2022-10-12

**Authors:** Konrad Lohse, Lars Höök, Karin Näsvall, Niclas Backström, Wellcome Sanger Institute Tree of Life programme, Wellcome Sanger Institute Scientific Operations: DNA Pipelines collective, Tree of Life Core Informatics collective, Darwin Tree of Life Consortium

**Affiliations:** 1 Institute of Evolutionary Biology, University of Edinburgh, Edinburgh, UK; 2 Evolutionary Biology Program, Department of Ecology and Genetics (IEG), Uppsala University, Uppsala, Sweden

**Keywords:** Leptidea sinapis, wood white, butterfly, genome sequence, chromosomal, Lepidoptera

## Abstract

We present a genome assembly from an individual male *Leptidea sinapis* (the wood white; Arthropoda; Insecta; Lepidoptera; Pieridae). The genome sequence is 686 megabases in span. The majority (99.99%) of the assembly is scaffolded into 48 chromosomal pseudomolecules, with three Z sex chromosomes assembled. Gene annotation of this assembly on Ensembl has identified 14,800 protein coding genes.

## Species taxonomy

Eukaryota; Metazoa; Ecdysozoa; Arthropoda; Hexapoda; Insecta; Pterygota; Neoptera; Endopterygota; Lepidoptera; Glossata; Ditrysia; Papilionoidea; Pieridae; Dismorphiinae; *Leptidea*; *Leptidea sinapis* (Linnaeus, 1758) (NCBI:txid189913).

## Background

The wood white butterfly ( *Leptidea sinapis*) is recognized by its white wings with dark apical spots on the forewings and its distinctively slow flight ( Thomas & Lewington, 2016). The preferred habitats are forest openings and meadows where herbaceous host plants from the family Fabaceae are present ( Friberg *et al.,* 2008; Wiklund, 1977). The distribution range covers a major part of the western Palearctic, the African continent excluded. Within Britain and Ireland, wood whites are restricted to fragmented, sheltered areas in southern Wales and England and a small region around Burren in western Ireland ( Thomas & Lewington, 2016). As a consequence of considerable population declines over the last decades, the wood white was included in the UK Biodiversity Action Plan in 2007, but the species has likely been under-surveyed ( Jeffcoate & Joy, 2011).

The wood white has long been the subject of ecological studies, investigating, for example, interaction with recently discovered cryptic and sympatric sister species and habitat preference variation ( Friberg *et al., * 2008; Friberg & Wiklund, 2009; Wiklund, 1977). Due to the presence of a striking chromosome number cline across the distribution range ( Dincă *et al.,* 2011; Lukhtanov *et al.,* 2020), the wood white has also developed into a model species for understanding the mechanistic underpinnings and evolutionary consequences of rapid karyotype evolution ( Lukhtanov *et al.,* 2020; Šíchová *et al.,* 2015; Talla *et al.,* 2019). Previous genomic and cytogenetic research have revealed a drastically expanded and unusually repeat-rich genome compared to most studied butterflies ( Talla *et al.,* 2017), and the presence of an unexpected sex-chromosome system ( Šíchová *et al.,* 2015). Existing genomic resources have also paved way for investigating, for example, the genetic basis of local adaptation ( Leal *et al.,* 2018; Näsvall *et al.,* 2021) and expression dynamics of sex-linked and autosomal genes ( Höök *et al.,* 2019). We foresee that the Darwin Tree of Life assembly presented here will be an important tool for forthcoming research on chromosome number dynamics, the association between structural rearrangements and reproductive isolation, the genetic basis of adaptive traits and the mechanistic underpinnings of microevolutionary processes in butterflies.

## Genome sequence report

**Figure 1.**
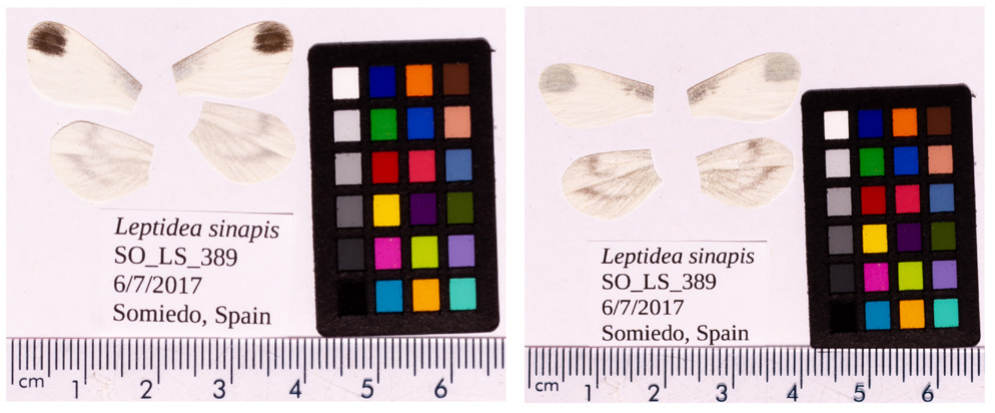
Fore and hind wings of the *Leptidea sinapis* specimen from which the genome was sequenced. Dorsal (left) and ventral (right) surface view of wings from specimen SO_LS_389 (ilLepSina1) from Asturias, Spain, used to generate Pacific Biosciences, 10X genomics and Hi-C data.

The genome was sequenced from a single male *L. sinapis* ( Figure 1) collected from Somiedo, Pigueces, Asturias, Spain (latitude 43.1489, longitude -6.3127). A total of 36-fold coverage in Pacific Biosciences single-molecule circular consensus (HiFi) long reads and 55-fold coverage in 10X Genomics read clouds were generated. Primary assembly contigs were scaffolded with chromosome conformation Hi-C data. Manual assembly curation corrected 20 missing/misjoins and removed 12 haplotypic duplications, reducing the assembly length by 1.30% and the scaffold number by 18.33%.

**Figure 2.**
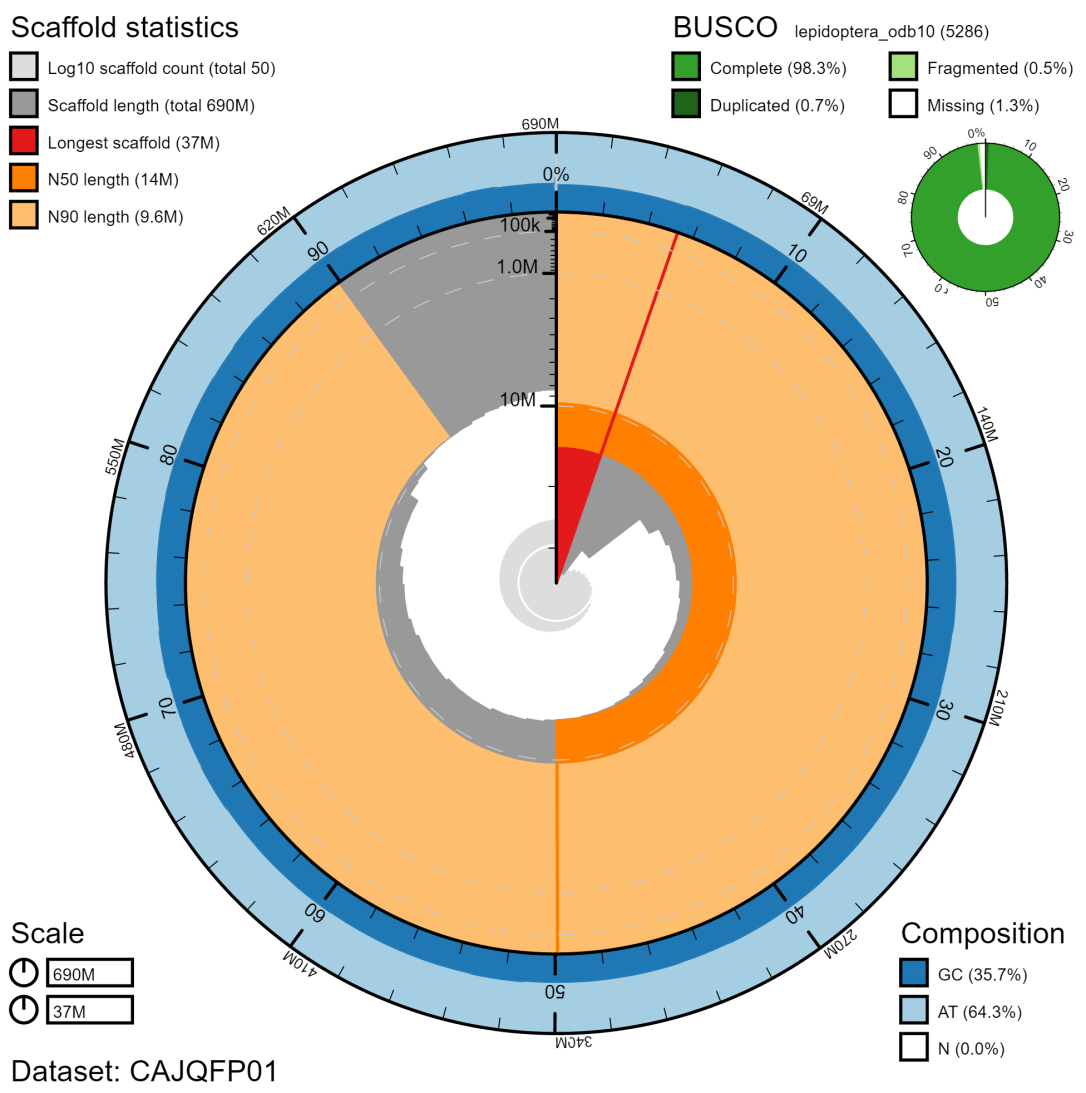
Genome assembly of *Leptidea sinapis*, ilLepSina1.1: metrics. The BlobToolKit Snailplot shows N50 metrics and BUSCO gene completeness. The main plot is divided into 1,000 size-ordered bins around the circumference with each bin representing 0.1% of the 685,599,024 bp assembly. The distribution of chromosome lengths is shown in dark grey with the plot radius scaled to the longest chromosome present in the assembly (36,552,532 bp, shown in red). Orange and pale-orange arcs show the N50 and N90 chromosome lengths (14,447,461 and 9,623,130 bp), respectively. The pale grey spiral shows the cumulative chromosome count on a log scale with white scale lines showing successive orders of magnitude. The blue and pale-blue area around the outside of the plot shows the distribution of GC, AT and N percentages in the same bins as the inner plot. A summary of complete, fragmented, duplicated and missing BUSCO genes in the lepidoptera_odb10 set is shown in the top right. An interactive version of this figure is available at https://blobtoolkit.genomehubs.org/view/ilLepSina1.1/dataset/CAJQFP01/snail.

The final assembly has a total length of 686 Mb in 49 sequence scaffolds with a scaffold N50 of 14.4 Mb ( Table 1). The majority, 99.99%, of the assembly sequence was assigned to 48 chromosomal-level scaffolds, representing 45 autosomes (numbered by sequence length) and three Z sex chromosomes ( Figure 2– Figure 5; Table 2). The assembly has a BUSCO v5.1.2 ( Manni *et al.,* 2021) completeness of 98.3% (single 97.6%, duplicated 0.7%) using the lepidoptera_odb10 reference set (n=5,286). While not fully phased, the assembly deposited is of one haplotype. Contigs corresponding to the second haplotype have also been deposited.

**Table 1.** Genome data for *Leptidea sinapis*, ilLepSina1.1.

*Project accession data*	
Assembly identifier	ilLepSina1.1
Species	*Leptidea sinapis*
Specimen	ilLepSina1 (genome assembly, Hi-C); ilLepSina2 (RNA-Seq)
NCBI taxonomy ID	189913
BioProject	PRJEB43801
BioSample ID	SAMEA7523467
Isolate information	Male, whole organism (ilLepSina1; ilLepSina2)
*Raw data accessions*	
PacificBiosciences SEQUEL II	ERR6565941
10X Genomics Illumina	ERR6054631-ERR6054634
Hi-C Illumina	ERR6054635
PolyA RNA-Seq Illumina	ERR6054636
*Genome assembly*	
Assembly accession	GCA_905404315.1
*Accession of alternate haplotype*	GCA_905404105.1
Span (Mb)	686
Number of contigs	62
Contig N50 length (Mb)	13.7
Number of scaffolds	49
Scaffold N50 length (Mb)	14.4
Longest scaffold (Mb)	34.3
BUSCO * genome score	C:98.3%[S:97.6%,D:0.7%], F:0.5%,M:1.3%,n:5,286

*BUSCO scores based on the lepidoptera_odb10 BUSCO set using v5.1.2. C= complete [S= single copy, D=duplicated], F=fragmented, M=missing, n=number of orthologues in comparison. A full set of BUSCO scores is available at https://blobtoolkit.genomehubs.org/view/ilLepSina1.1/dataset/CAJQFP01/busco.

**Figure 3.**
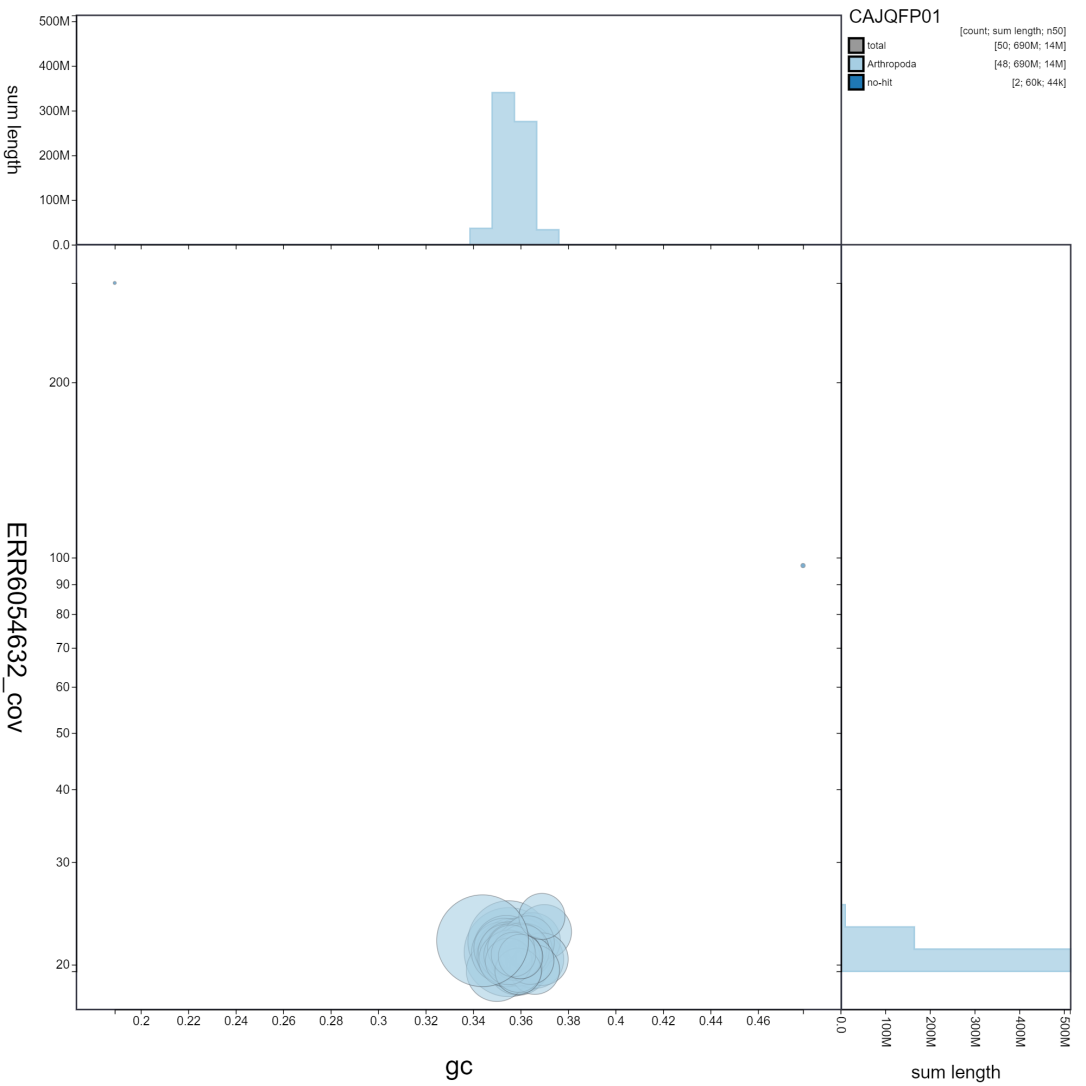
Genome assembly of *Leptidea sinapis*, ilLepSina1.1: GC coverage. BlobToolKit GC-coverage plot. Scaffolds are coloured by phylum. Circles are sized in proportion to scaffold length. Histograms show the distribution of scaffold length sum along each axis. An interactive version of this figure is available at https://blobtoolkit.genomehubs.org/view/ilLepSina1.1/dataset/CAJQFP01/blob.

**Figure 4.**
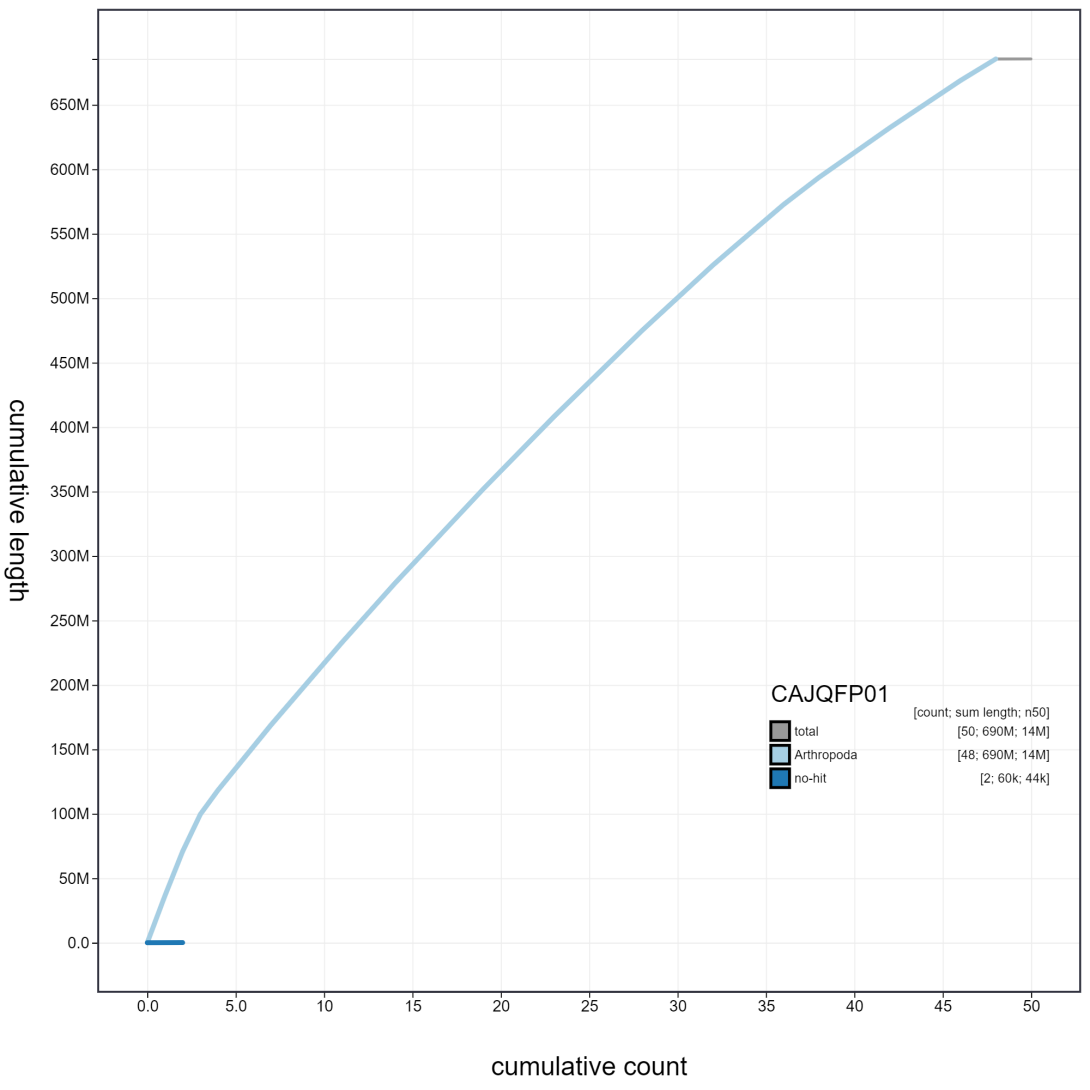
Genome assembly of *Leptidea sinapis*, ilLepSina1.1: cumulative sequence. BlobToolKit cumulative sequence plot. The grey line shows cumulative length for all scaffolds. Coloured lines show cumulative lengths of scaffolds assigned to each phylum using the buscogenes taxrule. An interactive version of this figure is available at https://blobtoolkit.genomehubs.org/view/ilLepSina1.1/dataset/CAJQFP01/cumulative.

**Figure 5.**
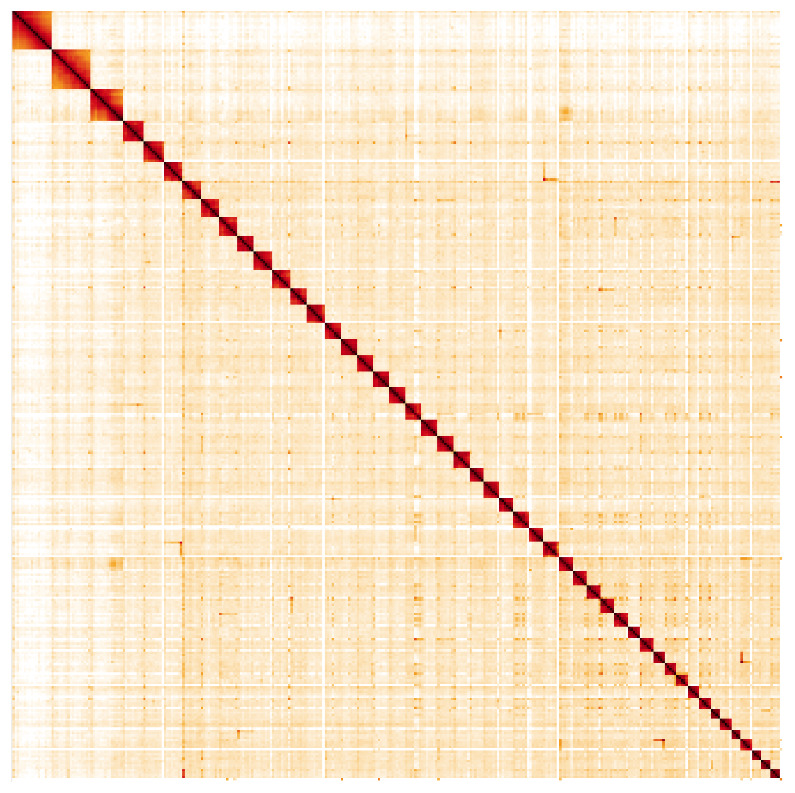
Genome assembly of *Leptidea sinapis*, ilLepSina1.1: Hi-C contact map. Hi-C contact map of the ilLepSina1.1 assembly, visualised in HiGlass. Chromosomes are arranged in size order from left to right and top to bottom. The interactive Hi-C map can be viewed at https://genome-note-higlass.tol.sanger.ac.uk/l/?d=aFf4d9rsTPefgcWgLKL1dA.

**Table 2.** Chromosomal pseudomolecules in the genome assembly of *Leptidea sinapis*, ilLepSina1.1. Chromosomes Z2 and Z3 are listed as chromosomes 2 and 3 with INSDC.

INSDC accession	Chromosome	Size (Mb)	GC%
FR990154.1	1	34.33	35.5
FR990155.1	Z2	28.77	35.5
FR990156.1	Z3	18.68	35.4
FR990157.1	4	17.20	35.6
FR990158.1	5	16.73	35.7
FR990159.1	6	16.62	36.0
FR990160.1	7	16.18	35.9
FR990161.1	8	15.95	35.0
FR990162.1	9	15.83	35.8
FR990163.1	10	15.81	35.4
FR990164.1	11	15.48	35.2
FR990165.1	12	15.36	35.3
FR990166.1	13	15.14	35.8
FR990167.1	14	14.85	36.0
FR990168.1	15	14.70	35.4
FR990169.1	16	14.65	35.9
FR990170.1	17	14.56	35.7
FR990171.1	18	14.45	35.6
FR990172.1	19	14.27	36.6
FR990173.1	20	14.10	35.8
FR990174.1	21	14.01	35.7
FR990175.1	22	13.72	35.5
FR990176.1	23	13.66	36.0
FR990177.1	24	13.61	35.7
FR990178.1	25	13.28	35.4
FR990179.1	26	13.27	36.5
FR990180.1	27	13.21	35.7
FR990181.1	28	12.90	35.4
FR990182.1	29	12.64	37.0
FR990183.1	30	12.62	36.0
FR990184.1	31	12.41	36.2
FR990185.1	32	12.01	36.3
FR990186.1	33	11.72	36.9
FR990187.1	34	11.70	36.0
FR990188.1	35	11.66	36.1
FR990189.1	36	10.56	35.5
FR990190.1	37	10.41	36.6
FR990191.1	38	9.73	35.9
FR990192.1	39	9.64	35.8
FR990193.1	40	9.62	36.4
FR990194.1	41	9.60	35.9
FR990195.1	42	9.39	35.6
FR990196.1	43	9.18	35.5
FR990197.1	44	9.06	35.9
FR990198.1	45	8.96	36.9
FR990199.1	46	8.39	35.7
FR990200.1	47	8.35	36.0
FR990153.1	Z1	36.55	34.4
FR990201.1	MT	0.02	18.9
-	Unplaced	0.04	47.9

## Genome annotation report

The ilLepSina1.1 genome was annotated using the Ensembl rapid annotation pipeline ( Table 1; https://rapid.ensembl.org/Leptidea_sinapis_GCA_905404315.1/). The resulting annotation includes 47,660 transcribed mRNAs from 14,800 protein-coding and 9,624 non-coding genes. There are 2.25 coding transcripts per gene and 8.29 exons per transcript.

## Methods

### Sample acquisition and nucleic acid extraction

Two male *L. sinapis* specimens (ilLepSina1, genome assembly, Hi-C; ilLepSina2, RNA-Seq) were collected from Somiedo, Pigueces, Asturias, Spain (latitude 43.1489, longitude -6.3127) using a net by Konrad Lohse, University of Edinburgh, who also identified the samples. The samples were frozen at -80°C.

DNA was extracted at the Scientific Operations Core, Wellcome Sanger Institute. The ilLepSina1 sample was weighed and dissected on dry ice with tissue set aside for Hi-C sequencing. Whole organism tissue was disrupted by manual grinding with a disposable pestle. Fragment size analysis of 0.01–0.5 ng of DNA was then performed using an Agilent FemtoPulse. High molecular weight (HMW) DNA was extracted using the Qiagen MagAttract HMW DNA extraction kit. Low molecular weight DNA was removed from a 200-ng aliquot of extracted DNA using 0.8X AMpure XP purification kit prior to 10X Chromium sequencing; a minimum of 50 ng DNA was submitted for 10X sequencing. HMW DNA was sheared into an average fragment size between 12–20 kb in a Megaruptor 3 system with speed setting 30. Sheared DNA was purified by solid-phase reversible immobilisation using AMPure PB beads with a 1.8X ratio of beads to sample to remove the shorter fragments and concentrate the DNA sample. The concentration of the sheared and purified DNA was assessed using a Nanodrop spectrophotometer and Qubit Fluorometer and Qubit dsDNA High Sensitivity Assay kit. Fragment size distribution was evaluated by running the sample on the FemtoPulse system.

RNA was extracted from whole organism tissue of ilLepSina2 in the Tree of Life Laboratory at the WSI using TRIzol, according to the manufacturer’s instructions. RNA was then eluted in 50 μl RNAse-free water and the RNA concentration assessed using a Nanodrop spectrophotometer and Qubit Fluorometer using the Qubit RNA Broad-Range (BR) Assay kit. Analysis of the integrity of the RNA was done using Agilent RNA 6000 Pico Kit and Eukaryotic Total RNA assay.

### Sequencing

Pacific Biosciences HiFi circular consensus and 10X Genomics read cloud DNA sequencing libraries were constructed according to the manufacturers’ instructions. Poly(A) RNA-Seq libraries were constructed using the NEB Ultra II RNA Library Prep kit. DNA and RNA sequencing was performed by the Scientific Operations core at the WSI on Pacific Biosciences SEQUEL II (HiFi), Illumina HiSeq X (10X) and Illumina HiSeq 4000 (RNA-Seq) instruments. Hi-C data were also generated from the whole organism of ilLepSina1 using the Arima v2 Hi-C kit and sequenced on an Illumina NovaSeq 6000 instrument.

### Genome assembly

Assembly was carried out with Hifiasm ( Cheng *et al.,* 2021); haplotypic duplication was identified and removed with purge_dups ( Guan *et al.,* 2020). One round of polishing was performed by aligning 10X Genomics read data to the assembly with longranger align, calling variants with freebayes ( Garrison & Marth, 2012). The assembly was then scaffolded with Hi-C data ( Rao *et al.,* 2014) using SALSA2 ( Ghurye *et al.,* 2019). The assembly was checked for contamination and corrected using the gEVAL system ( Chow *et al.,* 2016) as described previously ( Howe *et al.,* 2021). Manual curation ( Howe *et al.,* 2021) was performed using gEVAL, HiGlass ( Kerpedjiev *et al.,* 2018) and Pretext. The mitochondrial genome was assembled using MitoHiFi ( Uliano-Silva *et al.,* 2021), which performs annotation using MitoFinder ( Allio *et al.,* 2020). The genome was analysed and BUSCO scores generated within the BlobToolKit environment ( Challis *et al.,* 2020). Table 3 contains a list of all software tool versions used, where appropriate.

**Table 3.** Software tools used.

Software tool	Version	Source
Hifiasm	0.12	Cheng *et al.,* 2021
purge_dups	1.2.3	Guan *et al.,* 2020
SALSA2	2.2	Ghurye *et al.,* 2019
longranger align	2.2.2	https://support.10xgenomics.com/genome-exome/ software/pipelines/latest/advanced/other-pipelines
freebayes	1.3.1-17-gaa2ace8	Garrison & Marth, 2012
MitoHiFi	1	Uliano-Silva *et al.,* 2021
HiGlass	1.11.6	Kerpedjiev *et al.,* 2018
PretextView	0.1.x	https://github.com/wtsi-hpag/PretextView
BlobToolKit	2.6.4	Challis *et al.,* 2020

### Genome annotation

The Ensembl gene annotation system ( Aken *et al.,* 2016) was used to generate annotation for the *Leptidea sinapis* assembly ( GCA_905404315.1). Annotation was created primarily through alignment of transcriptomic data to the genome, with gap filling via protein-to-genome alignments of a select set of proteins from UniProt ( UniProt Consortium, 2019).

## Data Availability

European Nucleotide Archive: Leptidea sinapis (wood white). Accession number PRJEB43801; https://identifiers.org/ena.embl/PRJEB43801 ( Wellcome Sanger Institute, 2022) The genome sequence is released openly for reuse. The *L. sinapis* genome sequencing initiative is part of the Darwin Tree of Life (DToL) project. All raw sequence data and the assembly have been deposited in INSDC databases. Raw data and assembly accession identifiers are reported in Table 1.

## Author information

Members of the Wellcome Sanger Institute Tree of Life programme are listed here: https://doi.org/10.5281/zenodo.6866293.

Members of Wellcome Sanger Institute Scientific Operations: DNA Pipelines collective are listed here: https://doi.org/10.5281/zenodo.5746904.

Members of the Tree of Life Core Informatics collective are listed here: https://doi.org/10.5281/zenodo.6125046.

Members of the Darwin Tree of Life Consortium are listed here: https://doi.org/10.5281/zenodo.5638618.
