# Supplementary material for: The genome sequence of the wood white butterfly, Leptidea sinapis (Linnaeus, 1758)
Source: Wellcome Open Res. 2022 Oct 12;7:254. [Version 1] doi: 10.12688/wellcomeopenres.18118.1 (PMC10071140; doi:10.12688/wellcomeopenres.18118.1)
Supplement: Peer review history

# Peer review: Reviewer response for version 1

**DOI:** 10.21956/wellcomeopenres.20089.r54367

**Published:** 2023-04-03

**Authors:** Olli-Pekka Smolander

**Affiliations:** 1 Department of Chemistry and Biotechnology, Tallinn University of Technology, Tallinn, Estonia

The authors justify and describe well the production of the genome sequence of wood white butterfly and the related data. The procedure includes information on sampling, DNA isolation, sequencing methods as well as assembly and annotation. The results are described in good manner.

 The only point that I feel needs to be made is regarding the used software tools. It would be good if there was mention whether default parameters were used or if there were modifications to those.

Are sufficient details of methods and materials provided to allow replication by others?

Partly

Is the rationale for creating the dataset(s) clearly described?

Yes

Are the datasets clearly presented in a useable and accessible format?

Yes

Are the protocols appropriate and is the work technically sound?

Yes

Reviewer Expertise:

Genomics

I confirm that I have read this submission and believe that I have an appropriate level of expertise to confirm that it is of an acceptable scientific standard.

# Peer review: Reviewer response for version 1

**DOI:** 10.21956/wellcomeopenres.20089.r55069

**Published:** 2023-03-21

**Authors:** Rhys Parry

**Affiliations:** 1 The University of Queensland, Saint Lucia, Queensland, Australia

The data note presents a genome assembly of a male wood white butterfly, *Leptidea sinapis*, with a genome sequence of 686 megabases from Pacific Biosciences, 10X genomics and Hi-C data.

 The assembly is scaffolded into 48 chromosomal pseudomolecules, including three Z sex chromosomes, and gene annotation has identified 14,800 protein-coding genes. The methods are clearly defined, reproducible and appropriate.

 Given the species has been the subject of ecological studies and is a model species for understanding karyotype evolution, the genome assembly is expected to be a reasonable genomic resource for future butterfly evolution and adaptation research.

Are sufficient details of methods and materials provided to allow replication by others?

Yes

Is the rationale for creating the dataset(s) clearly described?

Yes

Are the datasets clearly presented in a useable and accessible format?

Yes

Are the protocols appropriate and is the work technically sound?

Yes

Reviewer Expertise:

Virology, genomics, genome assemblies, transcriptomics. Small RNA analysis and annotation.

I confirm that I have read this submission and believe that I have an appropriate level of expertise to confirm that it is of an acceptable scientific standard.
